# Germline mutations in candidate predisposition genes in individuals with cutaneous melanoma and at least two independent additional primary cancers

**DOI:** 10.1371/journal.pone.0194098

**Published:** 2018-04-11

**Authors:** Antonia L. Pritchard, Peter A. Johansson, Vaishnavi Nathan, Madeleine Howlie, Judith Symmons, Jane M. Palmer, Nicholas K. Hayward

**Affiliations:** Oncogenomics Group, QIMR Berghofer Medical Research Institute, Brisbane, Australia; University of Queensland Diamantina Institute, AUSTRALIA

## Abstract

**Background:**

While a number of autosomal dominant and autosomal recessive cancer syndromes have an associated spectrum of cancers, the prevalence and variety of cancer predisposition mutations in patients with multiple primary cancers have not been extensively investigated. An understanding of the variants predisposing to more than one cancer type could improve patient care, including screening and genetic counselling, as well as advancing the understanding of tumour development.

**Methods:**

A cohort of 57 patients ascertained due to their cutaneous melanoma (CM) diagnosis and with a history of two or more additional non-cutaneous independent primary cancer types were recruited for this study. Patient blood samples were assessed by whole exome or whole genome sequencing. We focussed on variants in 525 pre-selected genes, including 65 autosomal dominant and 31 autosomal recessive cancer predisposition genes, 116 genes involved in the DNA repair pathway, and 313 commonly somatically mutated in cancer. The same genes were analysed in exome sequence data from 1358 control individuals collected as part of non-cancer studies (UK10K). The identified variants were classified for pathogenicity using online databases, literature and *in silico* prediction tools.

**Results:**

No known pathogenic autosomal dominant or previously described compound heterozygous mutations in autosomal recessive genes were observed in the multiple cancer cohort. Variants typically found somatically in haematological malignancies (in *JAK1*, *JAK2*, *SF3B1*, *SRSF2*, *TET2* and *TYK2*) were present in lymphocyte DNA of patients with multiple primary cancers, all of whom had a history of haematological malignancy and cutaneous melanoma, as well as colorectal cancer and/or prostate cancer. Other potentially pathogenic variants were discovered in *BUB1B*, *POLE2*, *ROS1* and *DNMT3A*. Compared to controls, multiple cancer cases had significantly more likely damaging mutations (nonsense, frameshift ins/del) in tumour suppressor and tyrosine kinase genes and higher overall burden of mutations in all cancer genes.

**Conclusions:**

We identified several pathogenic variants that likely predispose to at least one of the tumours in patients with multiple cancers. We additionally present evidence that there may be a higher burden of variants of unknown significance in ‘cancer genes’ in patients with multiple cancer types. Further screens of this nature need to be carried out to build evidence to show if the cancers observed in these patients form part of a cancer spectrum associated with single germline variants in these genes, whether multiple layers of susceptibility exist (oligogenic or polygenic), or if the occurrence of multiple different cancers is due to random chance.

## Introduction

Cutaneous melanoma (CM) accounts for about 4% of skin cancers, but approximately 75% of deaths from the disease. CM results from the malignant transformation of melanocytes, the pigment-producing cells responsible for hair, eye and skin colour. CM risk is heritable and high penetrance germline mutations in *CDKN2A*, *CDK4*, *BAP1*, *MITF*, *TERT*, *POT1*, *ACD*, *TERF2IP* and *POLE* have been reported to contribute to CM development in some high density melanoma families [1, 2]. Additionally, genome-wide association studies (GWAS) have to date identified 20 low penetrance loci in non-high density familial (‘sporadic’) melanoma patients [3]. CM has a highly aberrant genome [4], strongly suggesting a role for aberrant DNA repair mechanisms. Notably, GWAS hits have been proximal to three genes involved in DNA repair, *ATM*, also an autosomal recessive cancer gene, (11q22-q23; rs73008229, genome-wide significance = 1.4x10^-12^), *PARP1* (1q42.12; rs3219090, genome-wide significance = 7.1x10^-12^) and *RAD23B* (9q31.2; rs10739221, genome-wide significance = 9.6x10^-9^) [3]; however the exact mechanisms behind risks associated with these GWAS hits have yet to be ascertained. Additionally, pathogenic variants in *BRCA1* and *BRCA2*, autosomal dominant cancer risk genes, both crucial in the process of homologous recombination DNA repair, increase risks to CM and uveal melanoma (UM), as well as several other cancer types including breast and ovarian cancer [5, 6]). The susceptibility to CM and UM associated with *BRCA1*/*BRCA2* is an example of melanotic tumours being part of a spectrum of tumours associated with well characterised cancer predisposition syndromes. Together, these data suggest a potential role for aberrations in DNA repair genes in the susceptibility to CM, UM and other cancers.

Recent evidence has shown an increased burden in pathogenic/probably pathogenic mutations in previously described ‘cancer’ genes (associated with autosomal dominant, autosomal recessive, cancer predisposition GWAS hits, or somatic driver events) in patients with paediatric cancer, compared to two control populations [7]. A second investigation recently examined ‘cancer predisposition’ mutation burden in a control population, unselected for cancer status. This study found that 3% of all variants identified were classified as deleterious and that 85% of all variants identified were of unknown significance; importantly, every individual carried multiple rare nonsynonymous variants in these genes, with an average of 68 variants/person (range:49–97) [8].

In our extensive collection of CM patients, a subset of 57 individuals had 2 or more additional independent primary tumours. These patients do not have genetic mutations in known high penetrance CM [1, 2] or UM (*BAP1* [9]) genes. The reason for the cancer accumulation in these individuals is therefore undetermined and could be a due to environmental components, random chance, polygenic risk, or rare unidentified high-risk cancer variants. Here, we hypothesise that mutations in a curated ‘cancer gene’ list and/or DNA repair genes are key elements to the cancer susceptibility in individuals with three or more primary cancers. We have therefore undertaken an investigation of 525 ‘cancer’ or DNA repair genes and describe the prevalence and spectrum of germline variants in this cohort of multiple cancer patients, compared to a control (non-cancer) cohort.

## Methods

### Study populations

The multiple cancer cases were all ascertained in Australia and were selected from those collected as part of the Q-MEGA project [10] which is a Queensland population-based study investigating the link between genetics and environment in melanoma development. Q-MEGA consists of four study samples: The Queensland Study of Childhood Melanoma (n = 101), The study of Melanoma in Adolescents (n = 298), The Study of Men over 50 (n = 178) and the Queensland Familial Melanoma Project (QFMP; n = 1897) [11]. Detailed information on personal and family cancer history was ascertained, which was used to identify individuals with at least three discrete primary cancers (one of which was CM and the other two were non-cutaneous) and without significant family history of CM; eligible individuals were those who did not have detectable deleterious mutations in *CDKN2A* or *CDK4* genes. This resulted in the identification of 57 individuals suitable for this study (S1 Table).

The total UK10K cohort consists of participants originating from 28 studies, broadly split into four categories 1. Population studies 2. Rare disease studies 3. Obesity studies and 4. Neurodevelopmental studies. Thirteen individual cohorts from across these categories are available for use as control populations, of which eight consist of unrelated individuals. In this study, only the UK10K cohorts consisting of unrelated individuals with permission to use as controls were assessed, which are: hypercholesterolemia (n = 125), thyroid (n = 121), severe insulin resistance (n = 121), rare neuromuscular diseases (n = 119), neurodevelopment disorders (n = 175), schizophrenia (n = 389 and n = 232) and autism spectrum disorder (n = 76); total cohort = 1358.

### Ethics and consent

All study participants gave written informed consent for participation. The multiple cancer cases were collected under ethics approval granted by the Human Research Ethics Committees of the QIMR Berghofer Medical Research Institute (HREC reference number: HREC/14/QPAH/495). The UK10K dataset is controlled by the “UK10K Data Access Committee”, who granted access to download the relevant cohorts under the conditions outlined in the Data Access Agreement and the Ethical Governance Framework.

### Sequencing and bioinformatic analysis

The 57 individuals with multiple cancers included in this study were analysed by WGS or WES. Sequencing was performed by Macrogen (Korea) on the Illumina Hiseq 2000 platform. Paired-end reads of 101bp were generated, with mean coverage of 60 to 96X. The BWA alignment algorithm was used to map sequence reads to the UCSC human genome reference build 19 [12]. SNVs were detected using bcftools and SAMtools mpileup with disabled BAQ computation [13] and indels were detected with pindel [14] and annotated to dbSNP144 by ANNOVAR [15]. Variants altering the coding sequence were selected that were present at a frequency of <1:100 (0.01) in the Kaviar aggregated control population [16]

### Selection of cancer predisposition and DNA repair genes

A total of 525 genes were selected for analysis, on the basis of the American College of Medical Genetics and Genomics (ACMG) gene list [17], the Online Mendelian Inheritance in Man (OMIM) [18], the LOVD database [19] and the literature [2, 7, 20–23]; S2 Table. This includes 65 autosomal dominant cancer predisposition genes, 31 autosomal recessive cancer predisposition genes, 23 genes encoding tyrosine kinases, 58 tumour suppressor genes, 232 cancer associated genes and 116 DNA repair genes.

### Validation of variants

The non-silent nucleotide substitution germline variant calls were assessed for read depth, reference count, alternative count and SNP call quality score. A variant quality score ≥70, alternative reads >2 and a ratio of alternative count:reference count ≥0.20 has been established as the criteria to maximize true variant calls and minimize false positives [21]. Sanger validation was performed on frameshift mutations in the multiple cancer cohort to ensure the correct base pairs were called for the in/del.

### Interpretation of variants

Variants were assessed using a number of methods in order to identify those that are pathogenic or potentially pathogenic. This included the assessment of variants present in ClinVar, ensuring the variant is correct (i.e. some genetic locations have multiple variants, with different functional consequences) and what level of review status has been established. Variants were assessed using four *in silico* tools that predict whether an amino acid alteration affects protein function: SIFT [24], PolyPhen-2 [25], likelihood ratio test [26] and Mutation Taster [27]. There were instances where a variant was not found / able to be assessed by a given *in silico* prediction tool, which are noted in the text where appropriate. Where consistent *in silico* prediction of deleterious effect of a SNV is present, this is indicative of a potentially damaging mutation. Curated publicly available germline variant databases were interrogated for evidence of prior mutation classification. The databases accessed were: LOVD [19] (APC, BRCA1, BRCA2, CBL, MLH1, MLH3, MSH2, MSH6, MUTYH, PMS2, RB1); UMD [28] (APC, MEN1, MLH1, MSH2, MSH6); IARC R18 [29] (TP53); ASU [30] (TERT); ARUP [31] (RET); NHGRI [https://research.nhgri.nih.gov/bic/] (BRCA1, BRCA2). Somatic mutation databases (COSMIC [32] and TCGA cBioPortal [33]) were assessed for hotspots that corresponded to germline mutations observed. Finally, OMIM and literature searches (e.g. NCBI PubMed) were used to examine the functional analyses performed on previously identified variants.

## Results

### Characteristics of the cohorts

**Fig 1:**
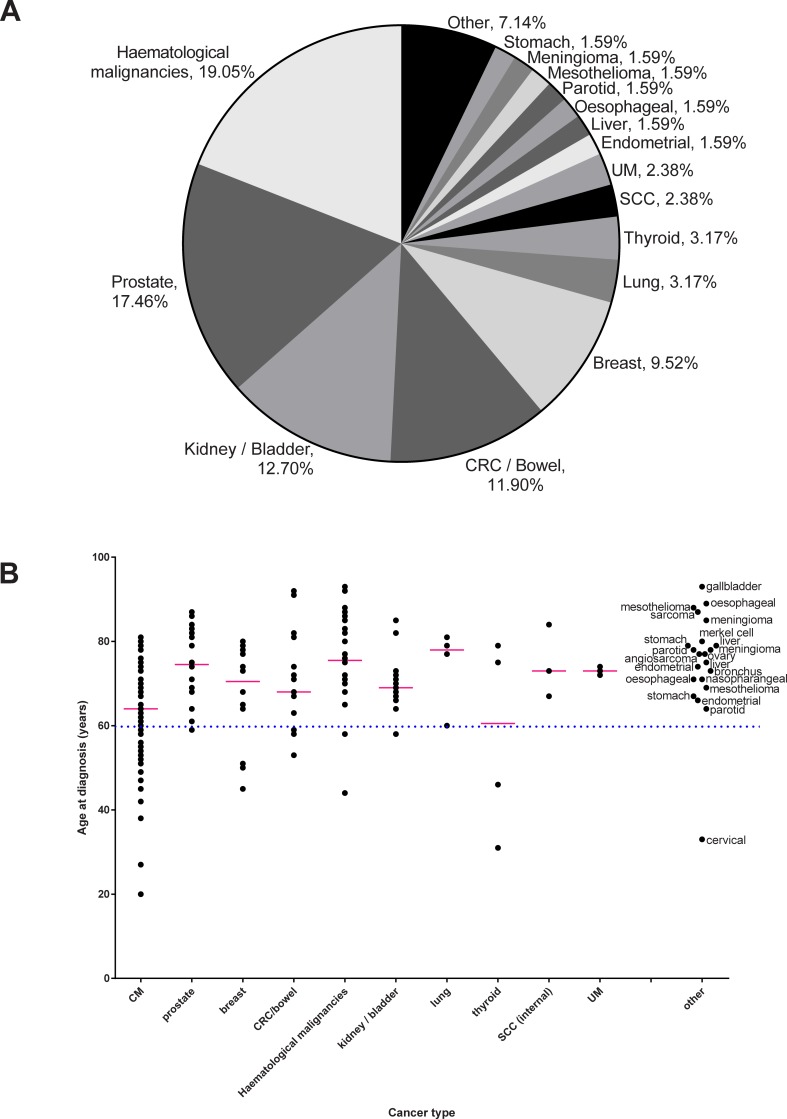
The cancer types and age at onset in the individuals with multiple independent primary cancers. A) Pie chart showing the frequency of cancer types in the total series of individuals with multiple independent primary cancer. All individuals were ascertained in Australia and were selected from those collected as part of the Q-MEGA project, a Queensland population-based study investigating the genetics of melanoma development; all individuals therefore presented with a history of cutaneous melanoma (CM) and are not represented in this figure. B) Age of onset for each of the cancers present in the multiple cancer patients. The dotted line at 60 years of age shows that the majority of cancers developed later in life. CRC: Colorectal cancer; UM: Uveal melanoma; SCC: Head and Neck Squamous Cell Carcinoma.

All of the individuals in the multiple cancer patient cohort had histopathologically confirmed CM and all cancers were registered at the Queensland Cancer Registry. The tumours present in this cohort are detailed in Fig 1A and the ages at diagnosis of the tumours are shown in Fig 1B and S1 Table. The majority of cancers were diagnosed later in life, but 25% of non-CM and 40% of CM were diagnosed at ≤60 years of age (Fig 1B). The median ages of the 1^st^ diagnosed cancer was 61 years, with the 2nd and 3rd cancers at 70 and 78 years, respectively. The UK10K cohort consists of several independently collected cohorts, with no information available on cancer in the individuals.

### Autosomal dominant genes

Of the 57 individuals with three or more distinct primary tumours, 53 instances of non-silent mutation were identified from the 63 autosomal dominant cancer predisposition genes at a Kaviar aggregate population frequency of less than 1:100 (47 missense, 1 splicing, 1 nonsense, and 4 non-frameshift ins/dels). Of these, 42 variants are present at a Kaviar aggregate population frequency of less than 1:2000 (33 missense, 1 splicing, 1 nonsense, and 2 non-frameshift in/dels); S3 Table. The nonsense mutation (CBL p.E658X) results in the termination of the protein product 249 amino acids prematurely and the removal of the vital tyrosinases at p. 700, 731 and 774, which are the key phosphorylation sites. It is likely, however, that this truncation does not result in a traditional oncogenic transformation of the CBL protein. Pathogenic mutations described to date require a functional tyrosine kinase binding domain (TKB, from p.51 – 349) and disruption of the α-helix formed between the TKB and RING domains; the truncating mutation in our cohort (p.Glu658X) does not disrupt this interaction [34]. Additionally, the RASopathy associated mutations cluster around the RING domain, with described mutations occurring from p.Q367–R420 [35], which is before the protein disruption described in our patient. Therefore, while this variant is not part of the known autosomal dominant cancer syndrome spectrum, the functional impact of this variant in terms of general cancer susceptibility is unknown. Of the 47 missense mutations observed, 1 was present in <1:2000 in the Kaviar population and predicted as damaging by all four *in silico* prediction algorithms (BRCA2 p.A75P rs28897701). The BRCA2 p.A75P mutation is, however, not classified as predisposing to breast/ovarian cancer by LOVD [19], or ClinVar. Two additional variants were present at a frequency of <1:100 individuals in Kaviar and predicted as damaging by *in silico* analysis. Additionally, a variant in APC (p.E2445D rs587782127) was predicted as damaging by the three algorithms able to assess it; this variant is classified as being of unknown significance by three submitters in ClinVar and the individual carrying the variant has not had colorectal cancer, commonly associated with *APC* germline mutations, to date.

The type and frequency of rare germline mutations in AD genes were then assessed in a control cohort (n = 1358); 1201 occurrences of non-silent mutation were identified from the 65 autosomal dominant cancer predisposition genes at a Kaviar aggregate population frequency of less than 1:100 (1132 missense mutations, 12 splicing, 5 nonsense, 18 frameshift and 34 non-frameshift ins/dels). Of these, 1077 variants are present at a Kaviar aggregate population frequency of less than 1:2000 (1005 missense, 15 splicing, 8 nonsense, 20 frameshift and 29 non-frameshift ins/del variants). Two of the nonsense mutations (p.C675X and p.E1013X) in *BRCA1*, 7 of the frameshift (p.N1626*11, p.N3024*16, p.Q1429*8, p.L2092*6, p.K1057*7 (x2), and p.F1546*21) in *BRCA2* and a single frameshift (p.Q60*6) in *PALB2* would predispose the carrier to breast/ovarian cancer. Finally of note, a p.Q12X variant in TP53 results in early protein truncation (full length protein is 394 amino acids long) and would result in Li-Fraumeni syndrome in the carrier. A total of 109 instances of missense variants of a frequency <1:2000 in the Kaviar population were predicted as damaging by all four prediction algorithms; of these, only one of which has been reported as pathogenic in ClinVar (RET p.I852M rs377767426 [36]). An additional 11 individuals had missense variants predicted as damaging by the three algorithms able to assess them, none of which are classified as pathogenic in ClinVar (S3 Table).

### Autosomal recessive genes

Examination of the autosomal recessive (AR) cancer predisposition gene variants from WES/WGS in all cohorts can only reveal where an individual has more than one variant in the same gene, but not whether they are on the same chromosome. In the multiple cancer case cohort, one individual had two missense variants in the same gene (*BRIP1*; S3 Table, highlighted yellow); neither of these variants were classified as damaging by all four prediction tools. In the UK10K cohort, 74 individual occurrences of more than one variant in the same AR gene were observed, covering 14 genes (*ATM*, *BRIP1*, *ERCC4*, *ERCC5*, *FANCA*, *FANCI*, *FANCM*, *MUTYH*, *RECQL4*, *SLCO2A1*, *WRN* and *XPC*, S3 Table, highlighted yellow). No individual had deleterious mutations observed more than once in the same gene, nor had more than one occurrence of a variant classified by ClinVar as pathogenic in the same gene.

It is plausible that heterozygous variant(s) in AR genes could cause a more subtle effect, such as inducing haploinsufficiency that increases susceptibility to cancer without causing an overt cancer syndrome. The frequencies of all variants in the AR genes are shown in S1A and S1B Fig. In the multiple cancer cases, truncating variants were seen in *FANCC* (p.R484X) and in *FANCF* (c.484/485 AG deletion). In the UK10K population, there are truncating mutations in 61 individuals, in 21 genes, including: *ATM*, *BRIP1*, *MUTYH*, *NBN*, *NTHL1*, *RAD51C*, *RECQL4*, *WRN*, *XPC*, members of the ERCC gene family (*ERCC1*, *ERCC3*, *ERCC5*) and members of the FANC gene family (*FANCA*, *FANCC*, *FANCD2*, *FANCF*, *FANCG*, *FANCI*, *FANCM*).

### Tumour suppressor genes

Tumour suppressor genes (TSG) play important roles in the control of neoplastic transformation and several have been discovered to be the source of AD cancer syndromes (such as *PTEN* and *TP53*). Examination of the TSG that are not otherwise classified as AD or AR cancer syndrome genes (n = 49) in the multiple cancer cohort revealed 50 missense, 1 nonsense, 2 frameshift and 6 non-frameshift in/del variants at a frequency of <1:100 in the Kaviar control population. At a frequency of <1:2000 individuals in Kaviar controls, there are 26 missense, 1 nonsense, 2 frameshift and 3 non-frameshift in/del variants. Of all the missense mutations, two variants were predicted as damaging by all tools (PMS1 p.T75I rs61756360 and TNFAIP3 p.R761H rs368859219) and another variant was predicted as damaging by the 3 tools that could assess it (TET2 p.I1873T rs116519313). None of the observed variants in TSGs have been classified as pathogenic by ClinVar. The most commonly mutated genes with a frequency <1:100 in Kaviar controls were CBFA2T3, NOTCH1 and TET2; those with a frequency <1:2000 are ARID1A, CAMTA1 and TET2. The nonsense variant (p.L737X; rs759242053) occurred in BUB1B. Variants in this gene can cause the AR disorder mosaic variegated aneuploidy, however, when a single deleterious mutation is present, this can result in a premature chromatid separation trait (OMIM entry 176430), which can lead to an increased susceptibility to tumour development. Two variants in TET2 are of note; the first, p.I1873T (rs116519313), is commonly reported as a somatic mutation (COSMIC ID = COSM41741 in haematopoietic/lymphocyte cancer x18); this patient had CM, colorectal cancer and mast cell leukaemia as distinct primary tumours and the second, an AT deletion at c.4874/4875, causing a frameshift at p.T1626, is in a patient with myeloproliferative disorder at age 65 years.

As described in S3 Table, in the UK10K cohort, 657 missense, 2 splicing, 14 nonsense, 13 frameshift and 13 non-frameshift variants were observed in TSGs at a frequency of <1:2000 in the Kaviar control population. Of these, 122 variants were classified as deleterious by all four prediction algorithms and none were currently classified as deleterious by ClinVar. The most commonly affected TSG in the UK10K cohort are *IGF2R*, *TET2*, *ARID1A* and *NOTCH1* at a frequency of <1:2000 in Kaviar control population. One of the nonsense mutations observed was in BUB1B (p.R770X; rs750364303), which as previously described could cause premature chromatid separation trait. A variant in ASXL1 (p.R693X rs373221034) has been reported to be somatically mutated 38 times in haematopoietic/lymphoid tissue/28 times in pancreatic cancer (ID = COSM51388) in the COSMIC database.

### Tyrosine kinase genes

Tyrosine kinases are commonly somatically mutated in tumours. Assessment of the location of variants in these revealed several locations that have been previously reported as somatic mutations (S3 Table). Of particular note in the multiple cancer case patients is the variant in JAK2 (p.V617F rs77375493), which is very highly somatically mutated in haematopoietic and lymphoid tissues (reported over 40,000 times in COSMIC, ID = COSM12600) and has been reported as a gain of function variant in myeloproliferative disorders [37], as well as acting as a predisposition variant in the germline [38]. The individual with this variant had myeloproliferative disorder at age 44. Additionally of potential functional impact: a frameshift variant in *JAK1* (c.3031insC) in an individual who had a history of CM (n = 2), lymphoma (at 75 years of age) and prostate cancer (at 83 years of age); a frameshift variant in TYK2 (c.1725-1728delinsTT), in an individual with a history of CM (at 42 years of age), lymphoma and clear cell renal carcinoma (both at 58 years of age), colorectal cancer (at 63 years of age) and prostate cancer (at 64 years of age); and a nonsense variant in ROS1 (p.L1209X) in an individual who had CM (at 63 years of age), stomach cancer (at 67 years of age), colorectal cancer (at 68 years of age), Merkel cell carcinoma (at 78 years of age) and thyroid cancer (at 79 years of age).

As shown in S3 Table, none of the variants in these kinase genes found in the UK10K control data have been reported as significantly mutated somatically in any cancer type. There are five frameshift variants (in *ABL1*, *ABL2* and *EGFR*) and five nonsense variants (in *JAK1*, *JAK2*, *PDGFRB* and *ROS1*) that would result in disruption of the protein product.

### ‘Other’ cancer genes

The final category of ‘cancer’ genes are those previously reported as playing an important role in cancer, but do not fit into the tumour suppressor or tyrosine kinase categories (S2 and S3 Tables). In the multiple cancer cases, several interesting variants are revealed, including in DNMT3A (p.R693H rs147001633 reported 121 times in haematopoietic/lymphoid tissue in COSMIC, ID = COSM442676), SF3B1 (p.K666N rs377023736 reported 31 times in haematopoietic/lymphoid tissue in COSMIC, ID = COSM132937) and SRSF2 p.P95L r751713049 reported 134 times in haematopoietic/lymphoid tissue in COSMIC, ID = COSM146288). The individual with the DNMT3A p.R693H variant had not had any haematological malignancy prior to death (at age 89 years), while the individual with the SF3B1 p.K666N variant had chronic myeloid leukaemia. A second individual, who had CM (at age 76 years), prostate cancer (at 86 years) and chronic myeloid leukaemia (at age 88 years), had a novel splice variant, 2bp into the intron after exon 18 of DNMT3A; this variant is of unknown functional consequence. The individual with the SRSF2 p.P95L variant is the same patient with the TET2 p.I1873T variant and mast cell leukaemia/colorectal cancer. None of the variants in the multiple cancer cases have been classified as pathogenic by ClinVar.

In the UK10K cohort, several variants are classified as pathogenic in ClinVar (S3 Table); however, none of these are associated with cancer predisposition by germline mutation. Two individuals in the UK10K control cohort had the same variant in DNMT3A (p.R693H) and two individuals had the same variant in SF3B1 (p.K666N) described in the multiple cancer cases. Additionally, an individual had the PIK3CA p.H1047L rs121913279 variant, which has been reported at high frequency in breast (n = 183), large intestine (n = 64) and endometrial (n = 43) cancers in COSMIC, ID = COSM776 and COSM94987.

### DNA repair genes

Many identified cancer predisposition genes encode DNA damage repair molecules; we have therefore additionally examined variants in genes not previously described as cancer genes, but which have a direct role in DNA damage repair. In the multiple cancer cases, there were 55 missense, 4 splicing, 6 nonsense, and 3 non-frameshift ins/del variants with a frequency of <1:100 in the Kaviar control population; of these, 31 missense, 2 splicing, 3 nonsense, and 1 non-frameshift ins/del variants had a frequency of <1:2000. A total of 6 missense variants at a frequency <1:2000 were predicted as damaging by all four algorithms, of which a single individual had two rare variants in WRNIP1 and another had a missense variant in POLE2 (p.L249I). The individual with two WRNIP missense variants (p.R537W rs145167237 and p.P615L rs372821009) had early onset cancers (Thyroid cancer at 31, CM at 42 and multifocal clear cell renal cancer at 58 years of age). Both of these variants are in the DNA-dependent ATPase and ssDNA annealing domain of the protein, which interacts with DNA polymerase δ, increasing the initiation frequency of DNA synthesis in response to DNA damage. The missense variant in POLE2 occurred in an individual who had colorectal cancer at age 59 years. None of the variants in DNA damage repair molecules were present in either ClinVar or COSMIC (at a frequency>5).

In the UK10K population, there were 781 missense, 17 splicing, 24 nonsense, 13 non-frameshift and 32 frameshift ins/del variants were present at a frequency of <1:2000 in the Kaviar controls. A total of 108 missense variants at a frequency <1:2000 were predicted as damaging by all four algorithms. None of these variants were in COSMIC (at a frequency>5). Two variants were present in ClinVar, both of which have been described in the literature in a compound heterozygote; the first in APTX in an individual with ataxia-ocular apraxia [39] and the second in LIG1 in an individual with an autosomal recessive immunodeficiency/DNA damage hypersensitivity syndrome [40].

### Global analysis of variants in cancer and DNA repair genes

The large number of variants of unknown significance in the datasets prompted a global comparison of the features of the variants, such as proportions of observed variants in our cohorts previously observed in the Kaviar control cohort, the frequencies of the types of mutation in the different gene classifications and the burden of variants present in each individual.

Exploration of the proportion of individuals from each cohort with variants previously observed in the Kaviar control population was carried out to assess whether there were a greater proportion of variants never/rarely previously observed in the Kaviar control cohort (n = 77,301) in the multiple cancer cases, compared to the UK10K population control cohort. No significant difference in the proportion of those variants observed <0.0005 (i.e. in less than 1:2000 chromosomes) in Kaviar in all genes assessed (Mann-Whitney *P* = 0.33; S2 Fig), i.e. there was not an over-representation of very rare/novel mutations in cancer/DNA repair genes in multiple cancer patients compared to an unselected cohort of individuals.

Examination of the types of mutation observed in each classification of gene showed that missense variants (predominantly of unknown significance) comprised the largest proportion of mutations observed. The frequency of damaging mutations (nonsense, frameshifts) were comparable between cohorts for autosomal dominant at frequencies of <1:100 (1.92% vs. 1.72%) or <1:2000 (2.6% vs. 2.38%) in Kaviar; S1A and S1B Fig. The frequencies of these damaging mutation types, however, were higher in the multiple cancer cases for genes classified as tumour suppressors (<1:100: 2.34% vs. 5.00% and <1:2000: 3.59% vs. 8.57%, respectively) and as tyrosine kinases (<1:100: 2.38% vs. 7.32% and <1:2000: 2.61 vs. 8.69%, respectively); S1A and S1B Fig.

**Fig 2:**
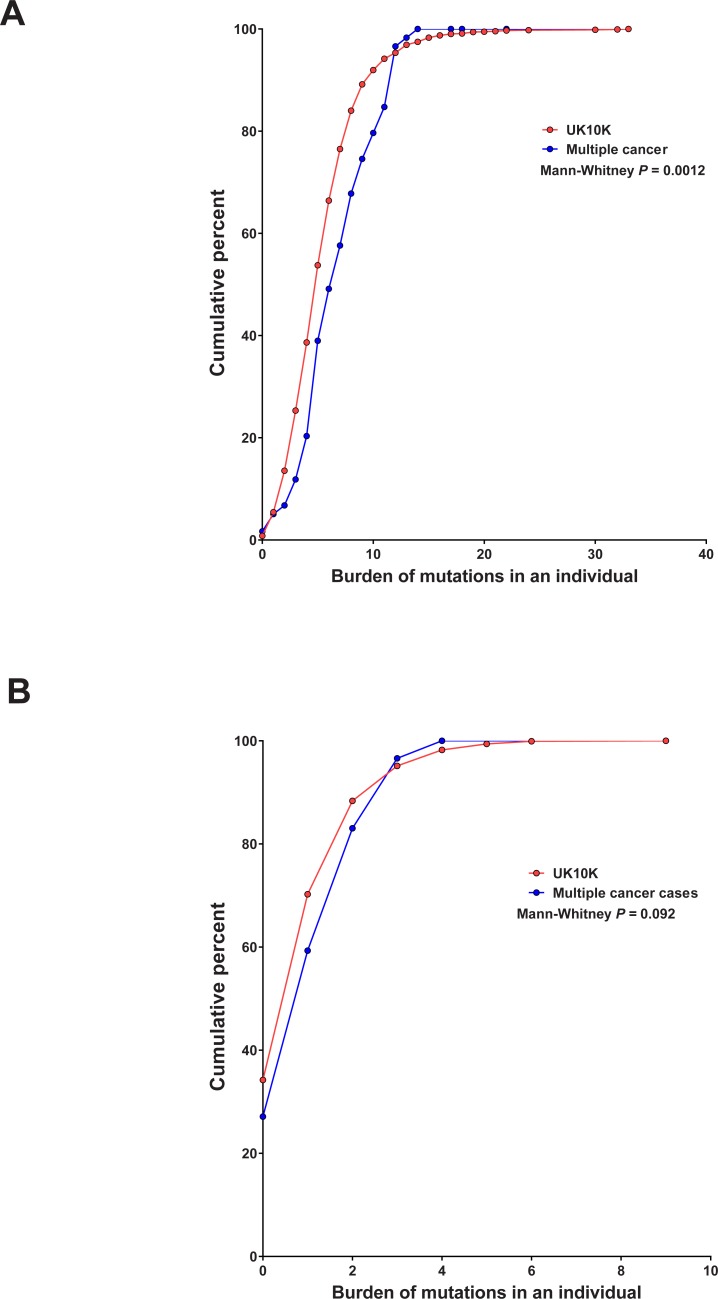
Cumulative percentage plots of variant burdens. The numbers of variants present at a frequency of <1:2000 in Kaviar in each of the gene classification lists (‘cancer’ genes and DNA repair genes) were calculated for each individual in the multiple cancer patients and the UK10K controls. The cumulative percentage of individuals with a given ‘burden’ of variants was then calculated and plotted for each cohort. A) The cumulative percentage plots of variant burden for all ‘cancer’ genes in the multiple cancer cases (blue) compared to the UK10K controls (red). B) The cumulative percentage plots of variant burden for DNA repair genes in the multiple cancer cases (blue) compared to the UK10K controls (red).

The numbers of mutations in these genes carried by each individual (i.e. the burden of mutations) at a frequency of <1:2000 in the Kaviar population was compared between people in the multiple cancer patient cohort to those in the UK10K cohort. This revealed that a greater number of multiple cancer cases carried multiple variants in cancer genes (Mann-Whitney *P* = 0.0012; Fig 2A) and in all genes combined (Mann-Whitney *P* = 0.0014), but not the DNA repair genes alone (Mann-Whitney *P* = 0.092; Fig 2B), compared to those in the UK10K control population.

## Discussion

There is somewhat anecdotal evidence that CM might be one of the cancer types associated with syndromes such as Li-Fraumeni Syndrome [41] and breast/ovarian cancer syndrome [6]. The low frequency of germline mutations in TP53 or BRCA1/2, respectively means that statistical evidence supporting these associations is rather weak. Further investigation is clearly required of CM being part of a previously described cancer syndrome. Accumulating evidence does however suggest that CM may be part of the ‘long tail’ of cancers which form rare components of the full spectrum of tumours associated with well characterised cancer predisposition syndromes. This study therefore aimed to investigate possible genetic predisposition to cancer in a cohort of patients with CM plus at least two additional independent primary tumour types, using a candidate gene approach. These genes were selected based on: a) function as known cancer predisposition genes (AD and AR), b) encoding tumour suppressors, c) encoding tyrosine kinases or d) DNA repair proteins (proteins of these latter subtypes are frequently aberrant in cancer) and e) those that are somatically relevant to cancer and not in these previous categories (‘other cancer genes’). In total, we examined coding region variants in 525 genes extracted from WES or WGS data.

In the AD gene category, there were few mutations of note in the multiple cancer cases. Given that the method of identification of these cancer cases was via their CM diagnosis and clinical follow-up, it was unlikely that individuals with an unidentified AD cancer syndrome would have been detected by our study. It is of interest that in the UK10K data, 2 deleterious variants were identified in *BRCA1* and 7 in *BRCA2* (a frequency of 0.15% and 0.52%, respectively); the estimated population frequency of pathogenic BRCA1/2 mutations is 1:800 (0.125%) to 1:1000 (0.1%) per gene, although the prevalence varies between ethnic groups and geographical areas [42]. A frequency of pathogenic variants in approximately 1:200 individuals for *BRCA2* is therefore higher than might be expected from a population of individuals selected for non-cancer studies. *MSH6* is a mismatch-repair gene involved in hereditary nonpolyposis colorectal cancer [43] and endometrial cancer [44]. In the UK10K cohort, 4 individuals had truncating mutations in *MSH6* (0.29%, approximately 1:350), which would cause an increase in colorectal cancer risk (by 8 times) and in endometrial cancer risk (26 times) more than the general population [45] in these individuals. Finally, truncating or previously functionally described deleterious missense mutations were observed in *CBL* (predisposing to Noonan syndrome, OMIM ID: 613563), *EPCAM* (Lynch syndrome/hereditary nonpolyposis colorectal cancer, OMIM ID: 613244), *NF1* (Neurofibromatosis, OMIM ID: 162200), *PALB2* (breast cancer, OMIM ID: 114480), *TP53* (Li Fraumeni Syndrome, OMIM ID: 151623) and *TSC2* (Tuberous sclerosis type 2, OMIM ID: 613254) in the UK10K control cohort. If we take these data as indicative of the types of deleterious genetic mutations present in a collection of individuals collated from non-cancer focused cohorts, it is clear that the multiple cancer cohort has a significant under-representation of such variants, and therefore no unidentified underlying cancer syndrome predisposition.

Similarly, we do not uncover a previously unrecognised AR cancer syndrome in our multiple cancer individuals. These cancer syndromes require homozygous or compound heterozygous mutations and often have a severe phenotype. It is however plausible that haploinsufficiency may play a role in increasing cancer susceptibility without causing an overt ablation of protein function leading to a cancer syndrome. In the UK10K cohort, there were a high number of truncating mutations in *FANCM* (n = 7), *MUTYH* (n = 7), *NTHL1* (n = 8) and *WRN* (n = 8), suggesting the proteins encoded by these genes can withstand the loss of one functional copy. In the multiple cancer patients, a nonsense variant in *FANCC* and a frameshift in *FANCF* were observed; three frameshifts were observed in these genes in the UK10 data. To date, no cancer susceptibility has formally been attributed to a heterozygous variant in an autosomal recessive cancer syndrome gene.

In the tumour suppressor, tyrosine kinase and ‘other’ categories of genes, several variants robustly described as somatic events in haematological malignancies were observed. As the JAK2 p.V617F, TET2 p.I1873T, SF3B1 p.K666N, SRSF2 p.P95L and DNMT3A p.R693H variants are frequent somatically mutated hotpots in haematological malignancy and have not been previously reported in the germline, it is plausible that our screen of buffy coat derived DNA detected somatic mutations. The participant with the JAK2 p.V617F variant previously had myeloproliferative disorder at age 44, and had their blood drawn for DNA extraction approximately 20 years later; at their death aged 85 years, had no reported diagnosis of recurrent haematological malignancy. The participants with a) the *SF3B1* p.K666N and b) *SRSF2* p.P95L/TET2 p.I1873T variants, respectively, both had haematological malignancies diagnosed in a closer timeframe after blood draw (exact date unknown) and therefore, tumour cells may have been detected. The variant in *DNMT3A* observed in haematological malignancies (p.R693H, as reported in COSMIC), was detected in an individual who had not developed such a tumour type prior to their death aged 89 years (previous cancers are: CM, prostate cancer and mesothelioma). While it is a common somatic driver event in haematological malignancy, this variant has also been described as an acquired event in healthy individuals and is present in ~4% of those over the age of 80 years [46]. It is clear from these findings that future screens of this nature require additional non-blood cell derived sources of DNA in order to further determine the true origin of the variants observed in these genes.

Frameshift or splice variants in JAK1, DNMT3A, TET2 and TYK2 all occurred in individuals with a history of CM and lymphoma/leukaemia and are not previously described as haematological malignancy hotspots; each of these individuals additionally had prostate and/or colorectal cancer, suggesting a potential phenotype associated with these variants.

Deleterious mutations in *BUB1B* are associated with premature chromatid separation, which can lead to increased susceptibility to cancer as well as to producing autosomal trisomy offspring, or other issues in conception [47]. A variant in *BUB1B* (p.L373X) occurred in an individual with CM, breast cancer and mesothelioma. *BUB1B* has been reported as being important in two of these tumours [48, 49], but germline variants have not been reported as being associated with these cancer types (OMIM ID: 602860). Finally, a truncating variant in *ROS1* (p.L1209X) was present in an individual who had CM, stomach, colorectal, merkel cell and thyroid cancers; *ROS1* has been somatically mutated in gastric and colorectal tumours [50], as well as spitzoid and acral melanomas [51, 52]; however, germline variants in this gene have only been associated with myocardial infarction (by GWAS [53]), not cancer.

Whether these variants additionally confer increased risk to the other malignancies in these individuals (which include cutaneous melanoma, colorectal cancer, clear cell renal carcinoma and prostate cancer), or there are further genetic predispositions in these individuals leading to the development of these independent primary tumours is an intriguing question. Also in these two classifications of genes, the frequency of damaging variants (frameshift in/dels and nonsense) were higher in the multiple cancer cases at frequencies of <1:100 and <1:2000 compared to the UK10K cohort. It is interesting that while these genes were previously associated with a specific type of cancer susceptibility, they could also confer risk of other primary tumour development and form part of a tumour spectrum; our observations therefore add further support to the previously observed similar results in prostate cancer [23] and in paediatric cancers [7] cohorts.

The final category of genes examined were those that encode DNA repair proteins. We observed a variant in POLE2, which was predicted as damaging by all *in silico* tools, in a patient who had colorectal cancer at age 59 years old. Variants in this gene have recently been associated with the development of colorectal cancer and polyposis [54, 55]. POLE2 is a subunit of the polymerase epsilon enzyme complex; we have previously demonstrated that a deleterious variant in another member of this complex, *POLE*, was associated with cutaneous melanoma development [56]. There is therefore an indication that variants in *POLE* and *POLE2* might be associated with susceptibility to multiple cancer types. Also of interest were two variants in *WRNIP1* that were predicted as damaging by all *in silico* tools, which occurred in the same individual, who had early onset cancers (thyroid cancer at 31, CM at 42 and multifocal clear cell renal cancer at 58 years of age. Both of these variants are present in the DNA-dependent ATPase and ssDNA annealing domain of the protein, which interacts with DNA polymerase δ, causing an increase in the initiation frequency of DNA synthesis in response to DNA damage. They are too far apart (1600bp) to infer phase from the sequencing data. There are a large number of missense (n = 22) and frameshift (n = 4) variants in WRNIP1 in the UK10K cohort, which could be suggestive of a degree of plasticity in the ability of the protein to withstand mutation.

The most intriguing implication from our observations is that there is a higher burden of variants in ‘cancer’ genes in patients with multiple primary cancers than in the control population. In the majority, these are missense variants of unknown significance. It is plausible that a number of rare mutations in different genes can act synergistically or additively together to increase susceptibility to cancer development. This potential mechanism by which the combination of variants leads to an increased susceptibility to cancer development is intriguing and would require very careful dissection and functional assessment, and perhaps with the advent of Cas9/CRISPR technology, this type of complex genetic manipulation might be more feasible in the future.

One of the possible reasons for the increased incidence in second and third cancers in these individuals may be due to treatment for previous malignancies, rather than a genetic predisposition. Both radiotherapy and chemotherapy have been shown to increase the likelihood of later development of haematological malignancies at between 5 and 8 years post-treatment. Close assessment of our data suggests this is likely not a significant confounding factor in these selected individuals. Firstly, 72% (n = 41) of the cohort had cutaneous melanoma as their first malignancy, for which treatment is was surgical excision. Of the 16 individuals who had a haematological malignancy, 8 were secondary to the CM, 3 were the first cancer and 5 followed a non-CM solid tumour (4 years, 10 years, 14 years 18 years and 19 years later); i.e. only one individual who developed a haematological malignancy falls into the predicted risk window.

Another possibility is that the increased burden in missense variants detected in the DNA of individuals with multiple primary cancers is as a somatic event as a consequence to the treatment of their previous tumour(s). Unfortunately, we only have DNA available from the buffy coat derived from blood cells, so cannot confirm in a second tissue (such as buccal swab) that variants are truly germline and not somatically acquired due to treatment. Comparison of the count of germline missense variants at a population frequency of <0.01 in all genes, except the cancer/DNA repair genes, revealed medians of 281 (interquartile range = 237–297) vs. 274 (interquartile range = 256–299) in the multiple cancer vs. UK10K cohorts, respectively; p>0.05). These data suggest that if treatment has altered the circulating lymphocyte DNA in the multiple cancer individuals, the effect in this cohort is subtle.

The UK10K cohort does not have cancer information available for the individuals included, so their treatment history is unknown. It is clear from the genetic data that there are individuals present in the UK10K cohort who have cancer syndromes caused by deleterious mutations (such as *BRCA1* nonsense, *BRCA2* frameshift and *APC* frameshift variants). This does, however, also indicate that the UK10K cohort is a representative cross-section of the general population and therefore any difference between this cohort and the multiple cancer cohort are potentially important.

It is apparent from this investigation that comprehensive automatic characterisation of mutations is still not currently feasible with each gene/mutation needing to be considered in its own right. This is due to several factors. The first of these being the way information is stored in ClinVar, which is by genomic position rather than by specific base pair alteration. An example of this is at chr2:47637479, rs63749984, in *MSH2*. This variant can either be a G>C transition (p.E205Q) or a G>T transition (p.E205X); it is the truncating mutation that is pathogenic (as reviewed by ClinVar panel of experts), while the missense mutation is of unknown significance (not present in ClinVar). This variant (rs63749984) and chromosomal location are therefore both currently classified as ‘pathogenic’ by ClinVar and without further scrutiny, the incorrect conclusion would be reached. A second important factor when establishing pathogenicity is the biology of the protein involved. For example, while the truncation observed in CBL (p.E658X) in the multiple cancer cohort and the frameshift in the UK10K cohort (a 7bp deletion at p.M222) could be automatically designated as damaging, there is no evidence in the literature of pathogenicity being conferred by truncation of CBL protein. Instead, oncogenic transformation requires disruption of the α-helix formed between the TKB and RING domains and an intact kinase domain. Currently, there is no way to perform high throughput assessment of such activating mutations, which require functional proof of oncogenic activation. Finally, it is clear that the predominant mutation type is missense ‘variant of unknown significance’. While these variants can be assessed using *in silico* prediction tools for impact on protein function, in depth experimental work is required in order to provide a more definitive conclusion about the nature of the mutation [57]. Therefore, high throughput *in silico* analysis of the missense variants is still largely limited to those that have been previously described as being deleterious.

## Conclusion

Given the observations of single pathogenic variants predisposing to multiple tumour types arising in distinct tissues, such as with BAP1 (uveal melanoma, mesothelioma, meningioma, clear cell renal cell carcinoma and cholangiocarcinoma [58, 59]), BRCA1 or BRCA2 (breast, ovarian, uveal melanoma) [60–62], it is plausible that other examples exist that have diverse effects that have yet to be described. Here, we approached this query with a cohort of patients who had three independent primary cancers to investigate a series of genes either previously associated with cancer, or which function in roles similar to those previously described as having a role in cancer. We identified a number of variants likely to have caused increased susceptibility to at least one of the primary tumours observed and have additionally shown an increased burden of mutation in affected individuals. Given the later age of onset of many of these tumours, it is plausible that these variants, either alone or in combination, do not have high impact on protein function and instead have more subtle cellular effects. Further investigations of this nature are clearly warranted. Similarly, other rationally selected gene sets could additionally be interrogated for their contribution to cancer predisposition. Given the increasing availability of germline data from cancer patients, studies such as these should be a research priority. The implication from this and other recent studies [7, 23] is that there are a significant number of germline genetic variations in genes known to be associated with cancer processes in individuals with a wide variety of tumour types.

## Supporting information

S1 Table Cancer information for the 57 individuals included in the multiple cancer cohort. Patients all had at least one cutaneous melanoma (CM) plus at least two independent cancer types. Where a patient had multiple CM, the age of 1st CM is given.
(DOCX)

S2 Table Information on the genes selected to make up the ‘cancer’ gene list and the DNA repair gene list. Tab 1: ‘cancer’ gene list, showing official gene symbol, classification of cancer gene, NCBI gene ID, cytogenetic location and a brief summary of encoded protein function and/or role in cancer.
Tab 2: DNA repair gene list, showing official gene symbol, NCBI gene ID, cytogenetic location and a brief summary of the encoded protein function.
(XLSX)

S3 Table UK10K control population and multiple cancer case population raw genetic data for each of the gene classification groups. (XLSX)

S1 Fig Frequency of different types of mutations in UK10K and multiple cancer cases, in each of the different gene classifications. A: For variants present at a frequency of <1:100 in Kaviar.
B: For variants present at a frequency of <1:2000 in Kaviar.
In/del = insertion/deletion mutation.
(PDF)

S2 Fig Assessment of whether the proportion variants never/rarely seen in the Kaviar control cohort was skewed in the multiple cancer cases (blue) or the UK10K cohort (red). To compare the distribution of types of mutations between the multiple cancer and the UK10K control cohort, we used a Monte Carlo version of a chi-squared test with 1,000,000 randomisations. P-values were adjusted for multiple test using the Benjamini-Hochberg procedure.
(PDF)

## References

[1] Read J, Wadt KA, Hayward NK. Melanoma genetics. J Med Genet. 2016;53(1):1–14. doi: 10.1136/jmedgenet-2015-103150 . 26337759 10.1136/jmedgenet-2015-103150

[2] Aoude LG, Wadt KA, Pritchard AL, Hayward NK. Genetics of familial melanoma: 20 years after CDKN2A. Pigment Cell Melanoma Res. 2015;28(2):148–60. doi: 10.1111/pcmr.12333 . 25431349 10.1111/pcmr.12333

[3] Law MH, Bishop DT, Lee JE, Brossard M, Martin NG, Moses EK, et al Genome-wide meta-analysis identifies five new susceptibility loci for cutaneous malignant melanoma. Nat Genet. 2015;47(9):987–95. doi: 10.1038/ng.3373 ; PubMed Central PMCID: PMC4557485. 26237428 10.1038/ng.3373 PMC4557485

[4] Cancer Genome Atlas N. Genomic Classification of Cutaneous Melanoma. Cell. 2015;161(7):1681–96. doi: 10.1016/j.cell.2015.05.044 . 26091043 10.1016/j.cell.2015.05.044 PMC4580370

[5] Mersch J, Jackson MA, Park M, Nebgen D, Peterson SK, Singletary C, et al Cancers associated with BRCA1 and BRCA2 mutations other than breast and ovarian. Cancer. 2015;121(2):269–75. doi: 10.1002/cncr.29041 ; PubMed Central PMCID: PMCPMC4293332. 25224030 10.1002/cncr.29041 PMC4293332

[6] Monnerat C, Chompret A, Kannengiesser C, Avril MF, Janin N, Spatz A, et al BRCA1, BRCA2, TP53, and CDKN2A germline mutations in patients with breast cancer and cutaneous melanoma. Familial cancer. 2007;6(4):453–61. doi: 10.1007/s10689-007-9143-y . 17624602 10.1007/s10689-007-9143-y

[7] Zhang J, Walsh MF, Wu G, Edmonson MN, Gruber TA, Easton J, et al Germline Mutations in Predisposition Genes in Pediatric Cancer. N Engl J Med. 2015;373(24):2336–46. doi: 10.1056/NEJMoa1508054 ; PubMed Central PMCID: PMC4734119. 26580448 10.1056/NEJMoa1508054 PMC4734119

[8] Bodian DL, McCutcheon JN, Kothiyal P, Huddleston KC, Iyer RK, Vockley JG, et al Germline variation in cancer-susceptibility genes in a healthy, ancestrally diverse cohort: implications for individual genome sequencing. PLoS One. 2014;9(4):e94554 doi: 10.1371/journal.pone.0094554 ; PubMed Central PMCID: PMC3984285. 24728327 10.1371/journal.pone.0094554 PMC3984285

[9] Aoude LG, Wadt K, Bojesen A, Cruger D, Borg A, Trent JM, et al A BAP1 mutation in a Danish family predisposes to uveal melanoma and other cancers. PLoS One. 2013;8(8):e72144 doi: 10.1371/journal.pone.0072144 ; PubMed Central PMCID: PMCPMC3747051. 23977234 10.1371/journal.pone.0072144 PMC3747051

[10] Baxter AJ, Hughes MC, Kvaskoff M, Siskind V, Shekar S, Aitken JF, et al The Queensland Study of Melanoma: environmental and genetic associations (Q-MEGA); study design, baseline characteristics, and repeatability of phenotype and sun exposure measures. Twin Res Hum Genet. 2008;11(2):183–96. doi: 10.1375/twin.11.2.183 ; PubMed Central PMCID: PMCPMC3677021. 18361720 10.1375/twin.11.2.183 PMC3677021

[11] Aitken J, Welch J, Duffy D, Milligan A, Green A, Martin N, et al CDKN2A variants in a population-based sample of Queensland families with melanoma. J Natl Cancer Inst. 1999;91(5):446–52. . 10070944 10.1093/jnci/91.5.446

[12] Li H, Durbin R. Fast and accurate short read alignment with Burrows-Wheeler transform. Bioinformatics. 2009;25(14):1754–60. doi: 10.1093/bioinformatics/btp324 ; PubMed Central PMCID: PMCPMC2705234. 19451168 10.1093/bioinformatics/btp324 PMC2705234

[13] Li H, Handsaker B, Wysoker A, Fennell T, Ruan J, Homer N, et al The Sequence Alignment/Map format and SAMtools. Bioinformatics. 2009;25(16):2078–9. doi: 10.1093/bioinformatics/btp352 ; PubMed Central PMCID: PMCPMC2723002. 19505943 10.1093/bioinformatics/btp352 PMC2723002

[14] Ye K, Schulz MH, Long Q, Apweiler R, Ning Z. Pindel: a pattern growth approach to detect break points of large deletions and medium sized insertions from paired-end short reads. Bioinformatics. 2009;25(21):2865–71. doi: 10.1093/bioinformatics/btp394 ; PubMed Central PMCID: PMCPMC2781750. 19561018 10.1093/bioinformatics/btp394 PMC2781750

[15] Wang K, Li M, Hakonarson H. ANNOVAR: functional annotation of genetic variants from high-throughput sequencing data. Nucleic Acids Res. 2010;38(16):e164 doi: 10.1093/nar/gkq603 ; PubMed Central PMCID: PMCPMC2938201. 20601685 10.1093/nar/gkq603 PMC2938201

[16] Glusman G, Caballero J, Mauldin DE, Hood L, Roach JC. Kaviar: an accessible system for testing SNV novelty. Bioinformatics. 2011;27(22):3216–7. doi: 10.1093/bioinformatics/btr540 ; PubMed Central PMCID: PMC3208392. 21965822 10.1093/bioinformatics/btr540 PMC3208392

[17] Green RC, Berg JS, Grody WW, Kalia SS, Korf BR, Martin CL, et al ACMG recommendations for reporting of incidental findings in clinical exome and genome sequencing. Genetics in medicine: official journal of the American College of Medical Genetics. 2013;15(7):565–74. doi: 10.1038/gim.2013.73 ; PubMed Central PMCID: PMC3727274. 23788249 10.1038/gim.2013.73 PMC3727274

[18] McKusick-Nathans Institute of Genetic Medicine JHUB, MD). Online Mendelian Inheritance in Man, OMIM® 2016 [cited 2016]. Available from: http://omim.org/.

[19] Fokkema IF, Taschner PE, Schaafsma GC, Celli J, Laros JF, den Dunnen JT. LOVD v.2.0: the next generation in gene variant databases. Human mutation. 2011;32(5):557–63. doi: 10.1002/humu.21438 . 21520333 10.1002/humu.21438

[20] Robles-Espinoza CD, Harland M, Ramsay AJ, Aoude LG, Quesada V, Ding Z, et al POT1 loss-of-function variants predispose to familial melanoma. Nat Genet. 2014;46(5):478–81. doi: 10.1038/ng.2947 ; PubMed Central PMCID: PMC4266105. 24686849 10.1038/ng.2947 PMC4266105

[21] Aoude LG, Pritchard AL, Robles-Espinoza CD, Wadt K, Harland M, Choi J, et al Nonsense mutations in the shelterin complex genes ACD and TERF2IP in familial melanoma. J Natl Cancer Inst. 2015;107(2). doi: 10.1093/jnci/dju408 . 25505254 10.1093/jnci/dju408 PMC4334787

[22] Yokoyama S, Woods SL, Boyle GM, Aoude LG, MacGregor S, Zismann V, et al A novel recurrent mutation in MITF predisposes to familial and sporadic melanoma. Nature. 2011;480(7375):99–103. Epub 2011/11/15. doi: 10.1038/nature10630 ; PubMed Central PMCID: PMC3266855. 22080950 10.1038/nature10630 PMC3266855

[23] Pritchard CC, Mateo J, Walsh MF, De Sarkar N, Abida W, Beltran H, et al Inherited DNA-Repair Gene Mutations in Men with Metastatic Prostate Cancer. N Engl J Med. 2016 doi: 10.1056/NEJMoa1603144 . 27433846 10.1056/NEJMoa1603144 PMC4986616

[24] Kumar P, Henikoff S, Ng PC. Predicting the effects of coding non-synonymous variants on protein function using the SIFT algorithm. Nat Protoc. 2009;4(7):1073–81. doi: 10.1038/nprot.2009.86 . 19561590 10.1038/nprot.2009.86

[25] Adzhubei I, Jordan DM, Sunyaev SR. Predicting functional effect of human missense mutations using PolyPhen-2. Curr Protoc Hum Genet. 2013;Chapter 7:Unit7 20. doi: 10.1002/0471142905.hg0720s76 ; PubMed Central PMCID: PMCPMC4480630. 23315928 10.1002/0471142905.hg0720s76 PMC4480630

[26] Chun S, Fay JC. Identification of deleterious mutations within three human genomes. Genome research. 2009;19(9):1553–61. doi: 10.1101/gr.092619.109 ; PubMed Central PMCID: PMCPMC2752137. 19602639 10.1101/gr.092619.109 PMC2752137

[27] Schwarz JM, Rodelsperger C, Schuelke M, Seelow D. MutationTaster evaluates disease-causing potential of sequence alterations. Nat Methods. 2010;7(8):575–6. doi: 10.1038/nmeth0810-575 . 20676075 10.1038/nmeth0810-575

[28] Beroud C, Hamroun D, Collod-Beroud G, Boileau C, Soussi T, Claustres M. UMD (Universal Mutation Database): 2005 update. Human mutation. 2005;26(3):184–91. doi: 10.1002/humu.20210 . 16086365 10.1002/humu.20210

[29] Bouaoun L, Sonkin D, Ardin M, Hollstein M, Byrnes G, Zavadil J, et al TP53 Variations in Human Cancers: New Lessons from the IARC TP53 Database and Genomics Data. Human mutation. 2016;37(9):865–76. doi: 10.1002/humu.23035 . 27328919 10.1002/humu.23035

[30] Podlevsky JD, Bley CJ, Omana RV, Qi X, Chen JJ. The telomerase database. Nucleic Acids Res. 2008;36(Database issue):D339–43. doi: 10.1093/nar/gkm700 ; PubMed Central PMCID: PMCPMC2238860. 18073191 10.1093/nar/gkm700 PMC2238860

[31] Margraf RL, Crockett DK, Krautscheid PM, Seamons R, Calderon FR, Wittwer CT, et al Multiple endocrine neoplasia type 2 RET protooncogene database: repository of MEN2-associated RET sequence variation and reference for genotype/phenotype correlations. Human mutation. 2009;30(4):548–56. doi: 10.1002/humu.20928 . 19177457 10.1002/humu.20928

[32] Forbes SA, Bindal N, Bamford S, Cole C, Kok CY, Beare D, et al COSMIC: mining complete cancer genomes in the Catalogue of Somatic Mutations in Cancer. Nucleic Acids Res. 2011;39(Database issue):D945–50. Epub 2010/10/19. gkq929 [pii]. doi: 10.1093/nar/gkq929 ; PubMed Central PMCID: PMC3013785. 20952405 10.1093/nar/gkq929 PMC3013785

[33] Cerami E, Gao J, Dogrusoz U, Gross BE, Sumer SO, Aksoy BA, et al The cBio cancer genomics portal: an open platform for exploring multidimensional cancer genomics data. Cancer Discov. 2012;2(5):401–4. doi: 10.1158/2159-8290.CD-12-0095 ; PubMed Central PMCID: PMCPMC3956037. 22588877 10.1158/2159-8290.CD-12-0095 PMC3956037

[34] Thien CB, Langdon WY. Cbl: many adaptations to regulate protein tyrosine kinases. Nature reviews Molecular cell biology. 2001;2(4):294–307. doi: 10.1038/35067100 . 11283727 10.1038/35067100

[35] Martinelli S, Stellacci E, Pannone L, D'Agostino D, Consoli F, Lissewski C, et al Molecular Diversity and Associated Phenotypic Spectrum of Germline CBL Mutations. Human mutation. 2015;36(8):787–96. doi: 10.1002/humu.22809 . 25952305 10.1002/humu.22809

[36] Demeester R, Parma J, Cochaux P, Vassart G, Abramowicz MJ. A rare variant, I852M, of the RET proto-oncogene in a patient with medullary thyroid carcinoma at age 20 years. Human mutation. 2001;17(4):354 doi: 10.1002/humu.42 . 11295841 10.1002/humu.42

[37] Passamonti F, Rumi E. Clinical relevance of JAK2 (V617F) mutant allele burden. Haematologica. 2009;94(1):7–10. doi: 10.3324/haematol.2008.001271 ; PubMed Central PMCID: PMCPMC2625431. 19118374 10.3324/haematol.2008.001271 PMC2625431

[38] Kilpivaara O, Mukherjee S, Schram AM, Wadleigh M, Mullally A, Ebert BL, et al A germline JAK2 SNP is associated with predisposition to the development of JAK2(V617F)-positive myeloproliferative neoplasms. Nat Genet. 2009;41(4):455–9. doi: 10.1038/ng.342 ; PubMed Central PMCID: PMCPMC3676425. 19287384 10.1038/ng.342 PMC3676425

[39] Moreira MC, Barbot C, Tachi N, Kozuka N, Uchida E, Gibson T, et al The gene mutated in ataxia-ocular apraxia 1 encodes the new HIT/Zn-finger protein aprataxin. Nat Genet. 2001;29(2):189–93. doi: 10.1038/ng1001-189 . 11586300 10.1038/ng1001-189

[40] Barnes DE, Tomkinson AE, Lehmann AR, Webster AD, Lindahl T. Mutations in the DNA ligase I gene of an individual with immunodeficiencies and cellular hypersensitivity to DNA-damaging agents. Cell. 1992;69(3):495–503. . 1581963 10.1016/0092-8674(92)90450-q

[41] Curiel-Lewandrowski C, Speetzen LS, Cranmer L, Warneke JA, Loescher LJ. Multiple primary cutaneous melanomas in Li-Fraumeni syndrome. Arch Dermatol. 2011;147(2):248–50. doi: 10.1001/archdermatol.2010.428 . 21339461 10.1001/archdermatol.2010.428

[42] Balmana J, Diez O, Rubio IT, Cardoso F, Group EGW. BRCA in breast cancer: ESMO Clinical Practice Guidelines. Ann Oncol. 2011;22 Suppl 6:vi31–4. doi: 10.1093/annonc/mdr373 . 21908500 10.1093/annonc/mdr373

[43] Miyaki M, Konishi M, Tanaka K, Kikuchi-Yanoshita R, Muraoka M, Yasuno M, et al Germline mutation of MSH6 as the cause of hereditary nonpolyposis colorectal cancer. Nat Genet. 1997;17(3):271–2. doi: 10.1038/ng1197-271 . 9354786 10.1038/ng1197-271

[44] Wijnen J, de Leeuw W, Vasen H, van der Klift H, Moller P, Stormorken A, et al Familial endometrial cancer in female carriers of MSH6 germline mutations. Nat Genet. 1999;23(2):142–4. doi: 10.1038/13773 . 10508506 10.1038/13773

[45] Baglietto L, Lindor NM, Dowty JG, White DM, Wagner A, Gomez Garcia EB, et al Risks of Lynch syndrome cancers for MSH6 mutation carriers. J Natl Cancer Inst. 2010;102(3):193–201. doi: 10.1093/jnci/djp473 ; PubMed Central PMCID: PMCPMC2815724. 20028993 10.1093/jnci/djp473 PMC2815724

[46] McKerrell T, Park N, Moreno T, Grove CS, Ponstingl H, Stephens J, et al Leukemia-associated somatic mutations drive distinct patterns of age-related clonal hemopoiesis. Cell Rep. 2015;10(8):1239–45. Epub 2015/03/04. doi: 10.1016/j.celrep.2015.02.005 ; PubMed Central PMCID: PMCPMC4542313. 25732814 10.1016/j.celrep.2015.02.005 PMC4542313

[47] Hanks S, Coleman K, Reid S, Plaja A, Firth H, Fitzpatrick D, et al Constitutional aneuploidy and cancer predisposition caused by biallelic mutations in BUB1B. Nat Genet. 2004;36(11):1159–61. doi: 10.1038/ng1449 . 15475955 10.1038/ng1449

[48] Roe OD, Anderssen E, Helge E, Pettersen CH, Olsen KS, Sandeck H, et al Genome-wide profile of pleural mesothelioma versus parietal and visceral pleura: the emerging gene portrait of the mesothelioma phenotype. PLoS One. 2009;4(8):e6554 doi: 10.1371/journal.pone.0006554 ; PubMed Central PMCID: PMCPMC2717215. 19662092 10.1371/journal.pone.0006554 PMC2717215

[49] Myrie KA, Percy MJ, Azim JN, Neeley CK, Petty EM. Mutation and expression analysis of human BUB1 and BUB1B in aneuploid breast cancer cell lines. Cancer Lett. 2000;152(2):193–9. . 10773412 10.1016/s0304-3835(00)00340-2

[50] Lee J, Lee SE, Kang SY, Do IG, Lee S, Ha SY, et al Identification of ROS1 rearrangement in gastric adenocarcinoma. Cancer. 2013;119(9):1627–35. doi: 10.1002/cncr.27967 . 23400546 10.1002/cncr.27967

[51] Wiesner T, He J, Yelensky R, Esteve-Puig R, Botton T, Yeh I, et al Kinase fusions are frequent in Spitz tumours and spitzoid melanomas. Nat Commun. 2014;5:3116 doi: 10.1038/ncomms4116 ; PubMed Central PMCID: PMCPMC4084638. 24445538 10.1038/ncomms4116 PMC4084638

[52] Couts KL, McCoach CE, Murphy D, Christiansen J, Turner J, Lewis KD, et al Acral Lentiginous Melanoma Harboring a ROS1 Gene Fusion With Clinical Response to Entrectinib. Precision Oncology. 2017;in press. doi: 10.1200/PO.16.00013 10.1200/PO.16.00013 35172482

[53] Shiffman D, Ellis SG, Rowland CM, Malloy MJ, Luke MM, Iakoubova OA, et al Identification of four gene variants associated with myocardial infarction. Am J Hum Genet. 2005;77(4):596–605. doi: 10.1086/491674 ; PubMed Central PMCID: PMCPMC1275608. 16175505 10.1086/491674 PMC1275608

[54] Spier I, Holzapfel S, Altmuller J, Zhao B, Horpaopan S, Vogt S, et al Frequency and phenotypic spectrum of germline mutations in POLE and seven other polymerase genes in 266 patients with colorectal adenomas and carcinomas. Int J Cancer. 2015;137(2):320–31. doi: 10.1002/ijc.29396 . 25529843 10.1002/ijc.29396

[55] Chubb D, Broderick P, Dobbins SE, Frampton M, Kinnersley B, Penegar S, et al Rare disruptive mutations and their contribution to the heritable risk of colorectal cancer. Nat Commun. 2016;7:11883 doi: 10.1038/ncomms11883 ; PubMed Central PMCID: PMCPMC4917884. 27329137 10.1038/ncomms11883 PMC4917884

[56] Aoude LG, Heitzer E, Johansson P, Gartside M, Wadt K, Pritchard AL, et al POLE mutations in families predisposed to cutaneous melanoma. Familial cancer. 2015;14(4):621–8. doi: 10.1007/s10689-015-9826-8 . 26251183 10.1007/s10689-015-9826-8

[57] Masica DL, Karchin R. Towards Increasing the Clinical Relevance of In Silico Methods to Predict Pathogenic Missense Variants. PLoS Comput Biol. 2016;12(5):e1004725 doi: 10.1371/journal.pcbi.1004725 ; PubMed Central PMCID: PMC4865359. 27171182 10.1371/journal.pcbi.1004725 PMC4865359

[58] Wadt K, Choi J, Chung JY, Kiilgaard J, Heegaard S, Drzewiecki KT, et al A cryptic BAP1 splice mutation in a family with uveal and cutaneous melanoma, and paraganglioma. Pigment Cell Melanoma Res. 2012;25(6):815–8. doi: 10.1111/pcmr.12006 . 22889334 10.1111/pcmr.12006 PMC7453745

[59] Wadt KA, Aoude LG, Johansson P, Solinas A, Pritchard A, Crainic O, et al A recurrent germline BAP1 mutation and extension of the BAP1 tumor predisposition spectrum to include basal cell carcinoma. Clinical genetics. 2014 doi: 10.1111/cge.12501 . 25225168 10.1111/cge.12501

[60] Hearle N, Damato BE, Humphreys J, Wixey J, Green H, Stone J, et al Contribution of germline mutations in BRCA2, P16(INK4A), P14(ARF) and P15 to uveal melanoma. Invest Ophthalmol Vis Sci. 2003;44(2):458–62. . 12556369 10.1167/iovs.02-0026

[61] Shattuck-Eidens D, McClure M, Simard J, Labrie F, Narod S, Couch F, et al A collaborative survey of 80 mutations in the BRCA1 breast and ovarian cancer susceptibility gene. Implications for presymptomatic testing and screening. JAMA. 1995;273(7):535–41. . 7837387

[62] Easton DF, Steele L, Fields P, Ormiston W, Averill D, Daly PA, et al Cancer risks in two large breast cancer families linked to BRCA2 on chromosome 13q12-13. Am J Hum Genet. 1997;61(1):120–8. ; PubMed Central PMCID: PMCPMC1715847. 9245992 10.1086/513891 PMC1715847

