# Supplementary material for: Germline mutations in candidate predisposition genes in individuals with cutaneous melanoma and at least two independent additional primary cancers
Source: PLoS One. 2018 Apr 11;13(4):e0194098. doi: 10.1371/journal.pone.0194098 (PMC5894988; doi:10.1371/journal.pone.0194098)
Supplement: S1 Table — Patients all had at least one cutaneous melanoma (CM) plus at least two independent cancer types. Where a patient had multiple CM, the age of 1st CM is given. (DOCX) [file pone.0194098.s001.docx]

Supplementary Table 1: Cancer information for the 57 individuals included in the cohort patients. Patients all had at least one cutaneous melanoma (CM) plus at least two independent cancer types. Where a patient had multiple CM, the age of 1^st^ CM is given

| **Sample ID** | **Cancer 1** | **Age** | **Cancer 2** | **Age** | **Cancer 3** | **Age** | **Cancer 4** | **Age** | **Cancer 5** | **Age** |
| --- | --- | --- | --- | --- | --- | --- | --- | --- | --- | --- |
| MCC_AUS1 | CM | 65 | UM | 72 | lymphoma | 72 |  |  |  |  |
| MCC_AUS2 | CM | 42 | thyroid papillary follicular | 31 | multifocal clear cell renal cancer | 58 |  |  |  |  |
| MCC_AUS3 | CM | 60 | CRC | 67 | liver | 79 |  |  |  |  |
| MCC_AUS4 | CM | 80 | renal clear cell | 68 | prostate | 69 |  |  |  |  |
| MCC_AUS5 | CM (x2) | 49 | CRC | 59 | liver | 75 |  |  |  |  |
| MCC_AUS6 | CM | 42 | breast | 64 | mesothelioma | 69 |  |  |  |  |
| MCC_AUS7 | CM | 60 | non-Hodgkins lymphoma | 68 | bronchus | 73 |  |  |  |  |
| MCC_AUS8 | CM | 71 | CLL | 71 | lymphoma small lymphocytic | 71 |  |  |  |  |
| MCC_AUS9 | CM | 38 | thyroid | 46 | prostate | 61 |  |  |  |  |
| MCC_AUS10 | CM (x2) | 63 | lymphoma | 75 | prostate | 83 |  |  |  |  |
| MCC_AUS11 | CM (x2) | 54 | breast | 73 | angiosarcoma | 77 |  |  |  |  |
| MCC_AUS12 | CM | 64 | prostate | 82 | polycythemia | 82 |  |  |  |  |
| MCC_AUS13 | CM | 61 | breast | 80 | meningioma | 85 |  |  |  |  |
| MCC_AUS14 | CM | 52 | CRC | 59 | systemic mastocytosis | 70 |  |  |  |  |
| MCC_AUS15 | CM | 68 | SCC vocal cord | 67 | UM | 73 |  |  |  |  |
| MCC_AUS16 | CM | 73 | UM | 74 | breast | 50 | B cell lymphoma | 70 |  |  |
| MCC_AUS17 | CM | 56 | bladder | 72 | CRC | 74 |  |  |  |  |
| MCC_AUS18 | CM (x5) | 51 | bladder | 67 | prostate | 68 |  |  |  |  |
| MCC_AUS19 | CM | 75 | prostate | 81 | B-cell lymphoma | 93 |  |  |  |  |
| MCC_AUS20 | CM | 63 | CRC | 68 | stomach | 67 | thyroid | 79 | merkel cell | 80 |
| MCC_AUS21 | CM (x5) | 45 | endometrial stromal sarcoma | 74 | thyroid follicular | 75 |  |  |  |  |
| MCC_AUS22 | CM | 60 | prostate | 59 | CRC | 59 | bladder | 64 |  |  |
| MCC_AUS23 | CM | 81 | prostate | 59 | breast | 77 | parotid spindle | 78 |  |  |
| MCC_AUS24 | CM | 42 | lymphoma - follicular | 58 | renal cell | 58 | CRC | 63 | prostate | 64 |
| MCC_AUS25 | CM | 62 | bilateral ovary | 77 | secretory meingioma | 78 |  |  |  |  |
| MCC_AUS26 | CM | 53 | cervical (tbc) | 33 | lung | 60 |  |  |  |  |
| MCC_AUS27 | CM | 68 | prostate | 74 | SCC parotid | 84 |  |  |  |  |
| MCC_AUS28 | CM | 55 | breast | 65 | multiple myeloma | 80 |  |  |  |  |
| MCC_AUS29 | CM (x2) | 55 | bladder TCC | 58 | CLL | 76 | NSCLC | 77 |  |  |
| MCC_AUS30 | CM | 61 | lymphoma | 70 | prostate | 71 | bowel | 81 |  |  |
| **Sample ID** | **Cancer 1** | **Age** | **Cancer 2** | **Age** | **Cancer 3** | **Age** | **Cancer 4** | **Age** | **Cancer 5** | **Age** |
| MCC_AUS31 * | CM (x2) | 73 | TCC kidney | 71 | TCC bladder | 73 |  |  |  |  |
| MCC_AUS32 * | CM | 76 | colon | 71 | myeloblastic syndrome | 75 |  |  |  |  |
| MCC_AUS33 | CM | 79 | prostate | 75 | lymphoma | 85 |  |  |  |  |
| MCC_AUS34 | CM | 69 | CRC | 58 | breast | 68 |  |  |  |  |
| MCC_AUS35 | CM | 67 | myeloproliferative disease | 65 | lymphoma | 92 | CRC | 92 |  |  |
| MCC_AUS36 | CM | 61 | breast | 79 | lung | 79 |  |  |  |  |
| MCC_AUS37 | CM (x2) | 59 | prostate | 69 | myeloma | 83 |  |  |  |  |
| MCC_AUS38 | CM | 69 | prostate | 79 | oesophageal | 89 |  |  |  |  |
| MCC_AUS39 | CM (x3) | 64 | parotid | 64 | lung | 81 |  |  |  |  |
| MCC_AUS40 | CM (x7) | 20 | breast | 51 | bowel | 53 |  |  |  |  |
| MCC_AUS41 | CM | 64 | lymphoma | 86 | prostate | 86 |  |  |  |  |
| MCC_AUS42 | CM | 58 | multiple myeloma | 72 | myeloid leukaemia | 77 |  |  |  |  |
| MCC_AUS43 | CM (x4) | 71 | CRC | 72 | gallbladder | 93 |  |  |  |  |
| MCC_AUS44 | CM | 64 | breast | 74 | myeloma | 87 |  |  |  |  |
| MCC_AUS45 | CM | 68 | bladder/urethra | 66 | myelodysplastic syndrome | 85 |  |  |  |  |
| MCC_AUS46 | CM | 74 | prostate | 81 | mesothelioma | 88 |  |  |  |  |
| MCC_AUS47 | CM | 64 | TCC bladder | ? | urethral | ? |  |  |  |  |
| MCC_AUS48 | CM | 64 | myeloproliferative | 44 | CRC | 82 |  |  |  |  |
| MCC_AUS49 | CM | 64 | prostate | 69 | stomach | 79 |  |  |  |  |
| MCC_AUS50 | CM | 70 | prostate | 84 | kidney | 85 |  |  |  |  |
| MCC_AUS51 | CM (x2) | 78 | prostate | 82 | bladder | 82 |  |  |  |  |
| MCC_AUS52 | CM | 76 | prostate | 86 | CML | 88 |  |  |  |  |
| MCC_AUS53 | CM | 74 | prostate | 87 | sarcoma | 87 |  |  |  |  |
| MCC_AUS54 | CM (x3) | 54 | prostate | 69 | bladder | 70 | oesophagus | 71 | nasopharangeal | 71 |
| MCC_AUS55 | CM (x8) | 47 | SCC (oral) | 73 | breast | 78 |  |  |  |  |
| MCC_AUS56 | CM | 27 | breast | 45 | endometrium | 66 |  |  |  |  |
| MCC_AUS57 | CM (x2) | 79 | TCC bladder | 69 | TCC kidney | 70 |  |  |  |  |

Abbreviations used in this table: chronic lymphocytic leukaemia (CLL), non-small cell lung carcinoma (NSCLC), squamous cell carcinoma (SCC), transitional cell carcinoma (TCC), uveal melanoma (UM)

* Husband and wife: unrelated to each other
