# Supplementary material for: Germline mutations in candidate predisposition genes in individuals with cutaneous melanoma and at least two independent additional primary cancers
Source: PLoS One. 2018 Apr 11;13(4):e0194098. doi: 10.1371/journal.pone.0194098 (PMC5894988; doi:10.1371/journal.pone.0194098)

**A**

# Frequency of types of mutations in UK10K and multiple cancer cases in variants <1:100 (<0.01) in Kaviar

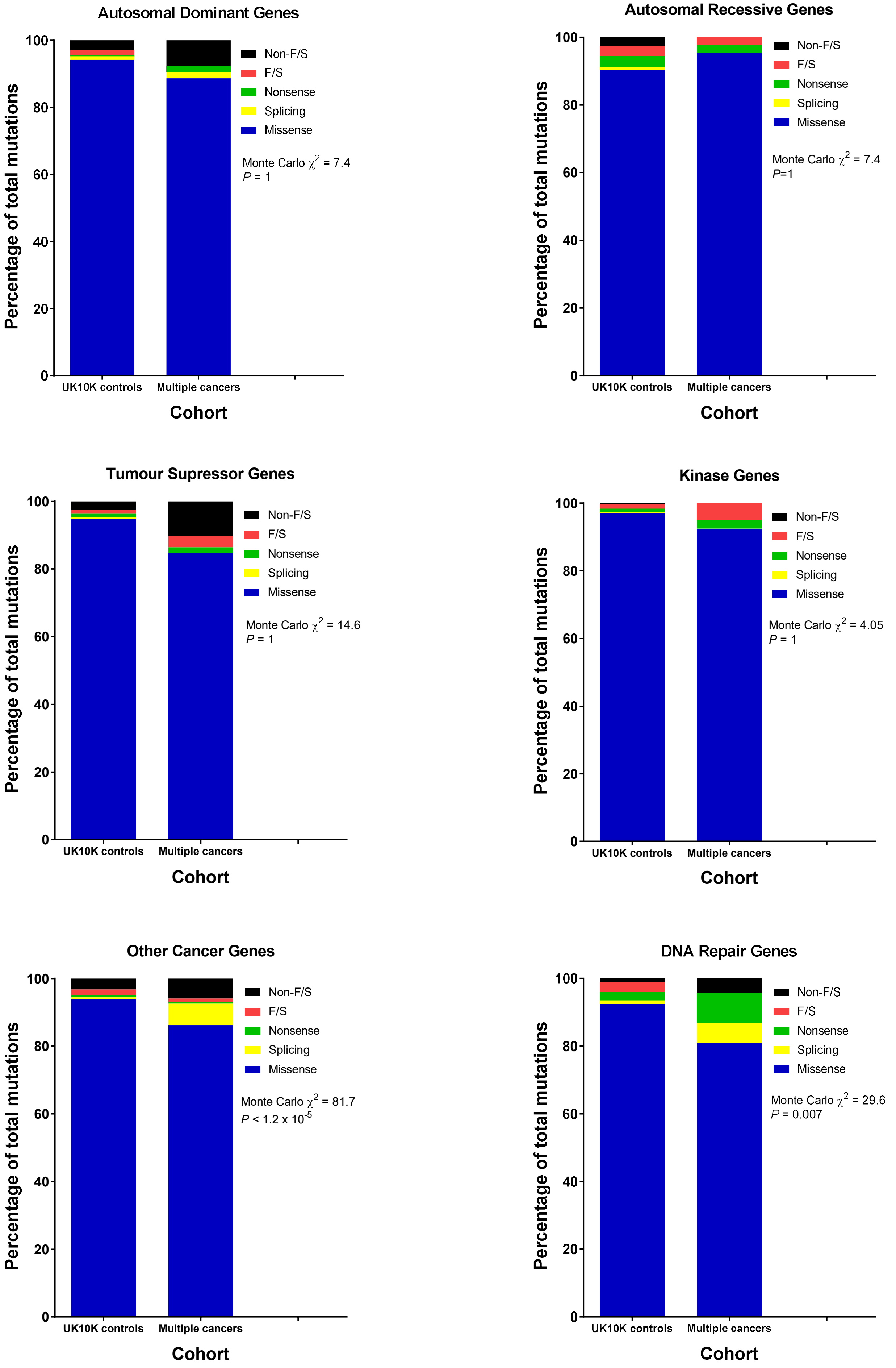

**B**

# Frequency of types of mutations in UK10K and multiple cancer cases in variants <1:2000 (<0.0005) in Kaviar

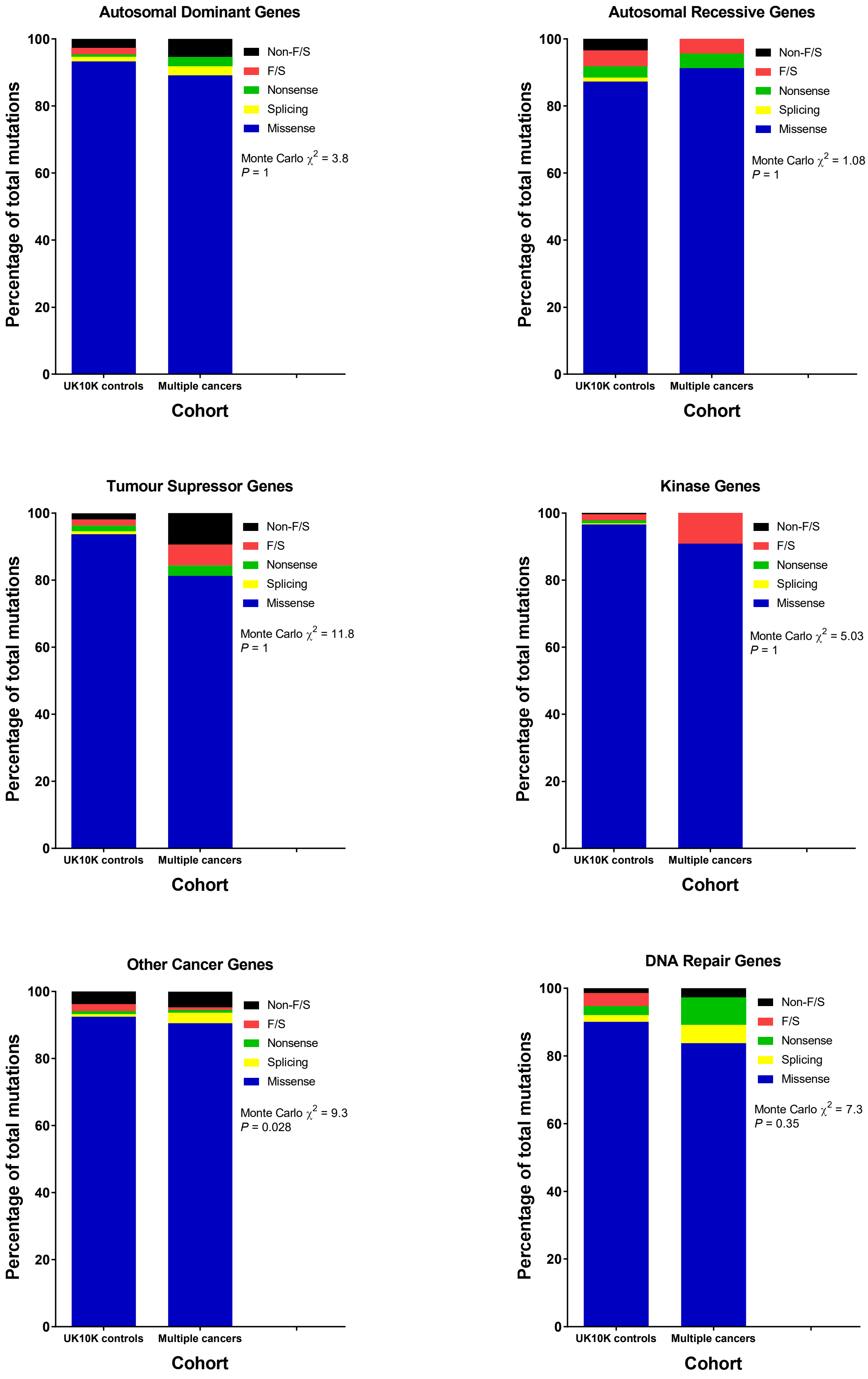

Supplement: S1 Fig — A: For variants present at a frequency of <1:100 in Kaviar. B: For variants present at a frequency of <1:2000 in Kaviar. In/del = insertion/deletion mutation. (PDF) [file pone.0194098.s004.pdf]
