# Supplementary figures and images for: Germline mutations in candidate predisposition genes in individuals with cutaneous melanoma and at least two independent additional primary cancers
Source: PLoS One. 2018 Apr 11;13(4):e0194098. doi: 10.1371/journal.pone.0194098 (PMC5894988; doi:10.1371/journal.pone.0194098)

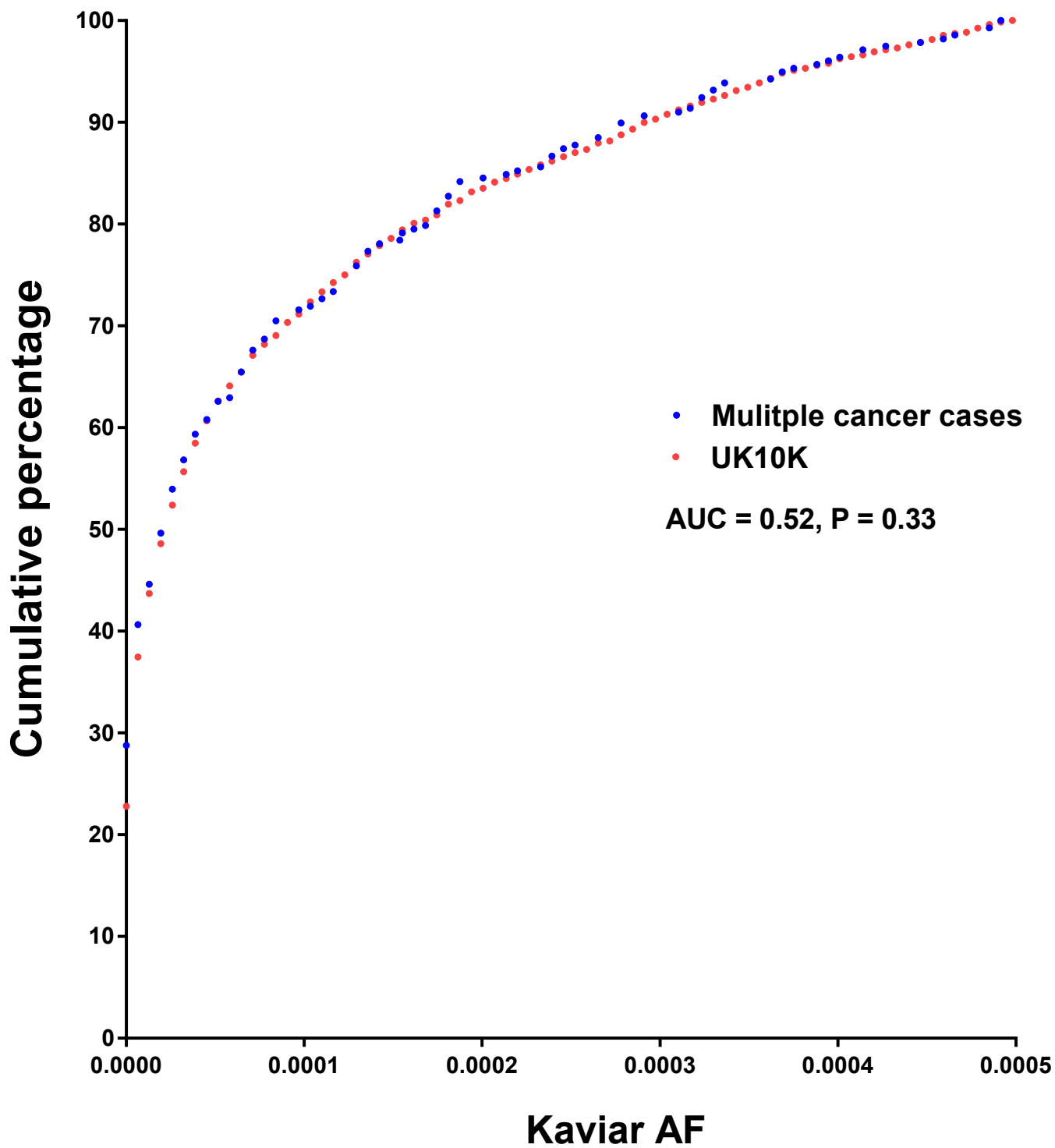

Supplement: S2 Fig — To compare the distribution of types of mutations between the multiple cancer and the UK10K control cohort, we used a Monte Carlo version of a chi-squared test with 1,000,000 randomisations. P-values were adjusted for multiple test using the Benjamini-Hochberg procedure. (PDF) [file pone.0194098.s005.pdf]
